# Radiation Dose and Fluoroscopy Time of Endovascular Treatment in Patients with Intracranial Lateral Dural Arteriovenous Fistulae

**DOI:** 10.1007/s00062-020-00982-3

**Published:** 2020-12-11

**Authors:** Robert Forbrig, Robert Stahl, Lucas L. Geyer, Yigit Ozpeynirci, Thomas Liebig, Christoph G. Trumm

**Affiliations:** Institute of Neuroradiology, University Hospital, LMU Munich, Marchioninistraße 15, 81377 Munich, Germany

**Keywords:** Coil embolization, LDAVF, Liquid embolization, Dose area product

## Abstract

**Purpose:**

Intracranial lateral dural arteriovenous fistula (LDAVF) represents a specific subtype of cerebrovascular fistulae, harboring a potentially life-threatening risk of brain hemorrhage. Fluoroscopically guided endovascular embolization is the therapeutic gold standard. We provide detailed dosimetry data to suggest novel diagnostic reference levels (DRL).

**Methods:**

Retrospective single-center study of LDAVFs treated between January 2014 and December 2019. Regarding dosimetry, the dose area product (DAP) and fluoroscopy time were analyzed for the following variables: Cognard scale grade, endovascular technique, angiographic outcome, and digital subtraction angiography (DSA) protocol.

**Results:**

A total of 70 patients (19 female, median age 65 years) were included. Total median values for DAP and fluoroscopy time were 325 Gy cm^2^ (25%/75% percentile: 245/414 Gy cm^2^) and 110 min (68/142min), respectively. Neither median DAP nor fluoroscopy time were significantly different when comparing low-grade with high-grade LDAVF (Cognard I + IIa versus IIb–V; *p* > 0.05, each). Transvenous coil embolization yielded the lowest dosimetry values, with significantly lower median values when compared to a combined transarterial/transvenous technique (DAP 290 Gy cm^2^ versus 388 Gy cm^2^, *p* = 0.031; fluoroscopy time 85 min versus 170 min, *p* = 0.016). A significant positive correlation was found between number of arterial feeders treated by liquid embolization and both DAP (r_s_ = 0.367; *p* = 0.010) and fluoroscopy time (rs = 0.295; *p* = 0.040). Complete LDAVF occlusion was associated with transvenous coiling (*p* = 0.001). A low-dose DSA protocol yielded a 20% reduction of DAP (*p* = 0.021).

**Conclusion:**

This LDAVF study suggests several local DRLs which varied substantially dependent on the endovascular technique and DSA protocol.

## Introduction

Intracranial dural arteriovenous fistulae (DAVF) are pathological shunts between meningeal arteries (i.e. fistula feeders, which commonly arise from branches of the external carotid artery) and intracranial venous sinus and/or cortical veins, accounting for 10–15% of intracranial vascular malformations [1]. The specific and relatively homogeneous subgroup of lateral DAVF (LDAVF) is usually located at the transverse and/or sigmoid sinus, is associated with sinus thrombosis or head trauma involving the temporal bone and commonly triggers pulsatile tinnitus [2, 3]. The LDAVFs can be categorized according to the Cognard classification into grades I–V [4], with a rising risk of intracranial hemorrhage in high-grade LDAVF (IIb–V) due to retrograde filling of cortical veins with (IIb) or without (III–V) sinus involvement. In this context, even though patients with a low-grade LDAVF (I or IIa) are not at risk of a bleeding, they commonly undergo treatment as the pulsatile tinnitus may substantially reduce the quality of life [3].

Fluoroscopically guided endovascular embolization is a standard first-line treatment in the field of intracranial LDAVF [2, 3]. The choice of the endovascular technique, e.g. transarterial liquid embolization with ethylene vinyl alcohol copolymer (EVOH) and/or transvenous coil embolization, strongly depends on the individual fistula morphology, particularly with respect to the accessibility of arterial fistula feeders and the type of venous drainage [2].

Since the national guidelines for radiation protection have been updated in 2018 [5], diagnostic reference levels (DRL) in endovascular procedures have gained increased attention. To date, the German DRLs for neuroradiological interventions are only defined for endovascular stroke and aneurysm treatment [6]. Further fluoroscopically guided neurointerventional procedures, such as embolization of LDAVFs, are not yet established.

In recent years, several authors published dosimetry data for neuroradiological interventions, including mechanical thrombectomy in acute stroke as well as endovascular treatment of intracranial aneurysms and arteriovenous malformations [7–16]; however, dosimetry data on the minimally invasive embolization of intracranial DAVFs are sparse [13–16]. Furthermore, these studies did not take the fistula subgroups (particularly LDAVF), the Cognard grade or the dedicated endovascular treatment technique (transarterial and/or transvenous embolization) into consideration.

In this retrospective single-center study we report detailed data on radiation dose and fluoroscopy time of endovascular treatment for patients with LDAVFs, considering the Cognard classification, endovascular technique, angiographic outcome and applied digital subtraction angiography (DSA) protocol. Our results may add valuable information for the establishment of novel DRLs in the field on intracranial DAVF treatment.

## Methods

**Fig. 1:**
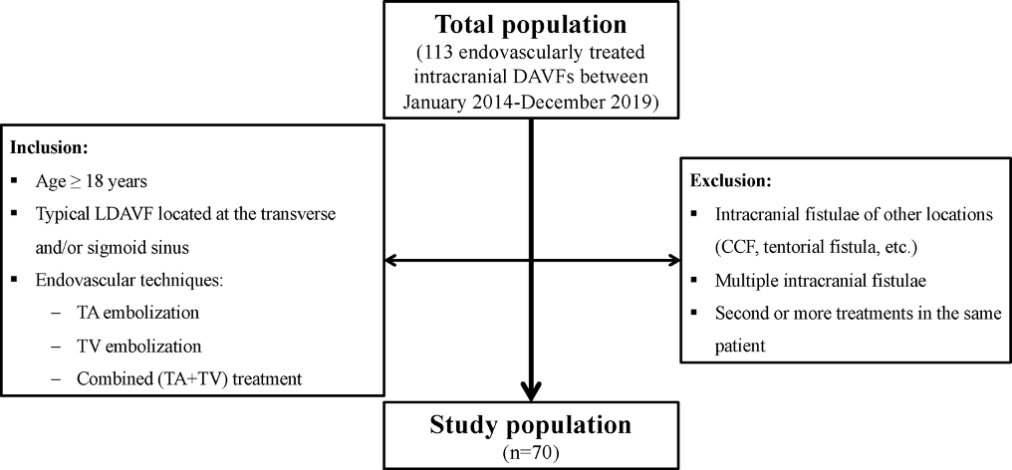
Flowchart of the inclusion and exclusion criteria. *CCF* carotid-cavernous fistula, *DAVF* dural arteriovenous fistula, *LDAVF* lateral dural arteriovenous fistula, *TA* transarterial, *TV* transvenous

We retrospectively analyzed all consecutive patients with a LDAVF who were endovascularly treated at our institution between January 2014 and December 2019. The following inclusion and exclusion criteria were defined (flowchart in Fig. 1):

Inclusion criteria:

- Age ≥18 years
- Typical LDAVF located at the transverse and/or sigmoid sinus, classified according to Cognard et al. into grades I–V [4]
- Endovascular techniques: transarterial (TA) embolization, transvenous (TV) embolization, combined (TA + TV) treatment.

Exclusion criteria:

- Intracranial fistulae of other locations (carotid-cavernous fistula, tentorial fistula, etc.)
- Multiple intracranial fistulae
- Second or more treatments in the same patient.

Endovascular procedures were performed with the patient under general anesthesia by 5 consultant interventional neuroradiologists with at least 7 years and up to over 20 years of experience in interventional neuroradiology, using a biplane angiographic unit (Axiom Artis dBA, Siemens Healthineers, Forchheim, Germany). The supra-aortic arteries and sinus/veins were accessed using a transfemoral approach. The nonionic iodinated contrast agent applied was Iomeprol 300 mg iodine/ml (Imeron®, Bracco Imaging Deutschland GmbH, Konstanz, Germany). The angiographic workflow comprised initial and final DSA acquisitions including arterial and venous phase on standard anteroposterior and lateral projections with a preferred field of view (FOV) of 32 cm and a frame rate of 1–4 f/s. Periprocedural DSA acquisitions were performed in arterial and early venous phase on working projections using a targeted FOV of 11 cm or 16 cm and an increased frame rate of up to 7.5 f/s for a sufficient visualization of the arteriovenous shunt. The frame rate of pulsed fluoroscopy was 7.5 f/s. With respect to the DSA acquisition type, two protocols were preset by the manufacturer as previously reported [7] and applied under discretion of the treating physicians:

- Low dose (LD): tube voltage 73 kV, pulse width 50 ms, dose 1820 µGy/pulse
- Normal dose (ND): tube voltage 73 kV, pulse width 100 ms, dose 3000 µGy/pulse

### Dosimetric Analysis

Imaging data and dose reports were retrieved from a dedicated picture archiving and communication system (PACS, Syngo Imaging, Siemens Healthineers) and reviewed by two neuroradiologists with 10 (R.F.) and 11 (C.G.T.) years of experience in interventional neuroradiology. The following parameters were recorded: LDAVF grades I–V according to the Cognard classification, endovascular technique (TA, TV, combined TA/TV embolization), angiographic outcome (complete versus incomplete occlusion, i.e. LDAVF downgrading), DSA acquisition count, DSA protocol (LD or ND), fluoroscopy time, and dose area product (DAP) associated with fluoroscopy (DAP_fluoroscopy_) and DSA (DAP_DSA_) acquisitions. The total DAP was calculated by summing DAP_fluoroscopy_ and DAP_DSA_. Dosimetry data were recorded by summing values of both X‑ray tubes.

### Statistics

Continuous data are provided as median (25%; 75% interquartile range), categorical data as counts and percent. Data of DAP and fluoroscopy time among the groups of Cognard grade, endovascular technique, angiographic outcome, and DSA protocol were initially assessed for normality with the Kolmogorov-Smirnov test. Based on these results we used pairwise nonparametric Mann-Whitney U-tests to evaluate intergroup differences and applied Bonferroni correction for multiple comparisons. Correlations between the two variables number of treated feeders and number of coils with radiation dose and fluoroscopy time were examined with the Spearman rank correlation coefficient and the Pearson correlation coefficient, respectively. Data analysis was performed using IBM SPSS Statistics for Windows, Version 25.0 (IBM, Armonk, NY, USA). A level of significance of *p* = 0.05 was used throughout the study.

## Results

### Patients

Baseline characteristics of endovascularly treated patients with intracranial LDAVF are summarized in Table 1. Within the study period, 70/113 (61.9%) patients with intracranial DAVFs met the inclusion and exclusion criteria. The median patient age was 65 years (range 25–80 years), 19/70 (27.1%) patients were women. Of the patients 65/70 (92.9%) underwent elective and 5/70 (7.1%) emergency endovascular treatment of LDAVF. Regarding the latter group, four patients suffered from LDAVF-associated intracranial hemorrhage (parenchymal bleeding, *n* = 3; subarachnoid bleeding, *n* = 1) and one patient from acute onset of trigeminal palsy accompanied by vertigo.

**Table 1:** Baseline characteristics of 70 endovascularly treated patients with LDAVF

**Median age**, years (range)	65 (25–80)	
**Gender, ***n* (%)		
Female	19/70 (27.1)	
Male	51/70 (72.9)	
**Indication**, *n* (%)		
*Elective*	*65/70 (92.9)*	
Pulsatile tinnitus	60/65 (92.3)	
Ipsilateral headache	8/65 (12.3)	
Vertigo	6/65 (9.2)	
*Emergency*	*5/70 (7.1)*	
ICH	3/5 (60.0)	
SAH	1/5 (20.0)	
Trigeminal palsy and vertigo	1/5 (20.0)	
**Cognard grade**, *n* (%)		
I	12/70 (17.1)	
IIa	4/70 (5.7)	
IIb	17/70 (24.3)	
III	10/70 (14.3)	
IV	26/70 (37.1)	
V	1/70 (1.4)	
**Endovascular technique/angiographic outcome**	**Complete occlusion (*n*** **=** **28)**	**Incomplete occlusion (*n*** **=** **42)**
*TA liquid embolization (n* *=* *49)*	13/49 (26.5%)	36/49 (73.5%)
*TV coil embolization (n* *=* *14)*	11/14 (78.6%)	3/14 (21.4%)
*Combined (n* *=* *7)*	4/7 (57.1%)	3/7 (42.9%)

Baseline characteristics of 70 endovascularly treated patients with LDAVF

*LDAVF* lateral dural arteriovenous fistula, *ICH* intracerebral hemorrhage, *SAH* subarachnoid hemorrhage, *TA* transarterial, *TV* transvenous

With respect to the Cognard classification, 12/70 (17.1%) patients presented with LDAVF grade I, 4/70 (5.7%) with grade IIa, 17/70 (24.3%) with grade IIb, 10/70 (14.3%) with grade III, 26/70 (37.1%) with grade IV, and 1/70 (1.4%) with grade V.

Of the patients 49/70 (70.0%) underwent TA treatment alone using a dedicated liquid embolic agent in each case (EVOH: Onyx®, Medtronic, Dublin, Ireland or Squid®, ab medica/Balt, Düsseldorf, Germany). In detail, 18/49 (36.7%) TA treated patients presented with a LDAVF Cognard grade I–IIb, and 31/49 (63.3%) with grade III–V. One arterial feeder was embolized in 26/49 (53.1%), two feeders in 13/49 (26.5%), three feeders in 9/49 (18.4%), and four feeders in 1/49 (2.0%) patients, 14/70 (20.0%) patients underwent sole TV embolization using coils in each case (median 15 coils; range 2–33 coils), including 3 patients with a LDAVF grades III–V, 7/70 (10.0%) patients were treated by a combination of TA liquid and TV coil embolization.

After the intervention, 28/70 (40.0%) and 42/70 (60.0%) LDAVFs were completely and incompletely occluded, respectively. In detail, incomplete occlusion (i.e., downgrading) was documented in 9/16 (56.3%) low-grade (Cognard I + IIa) and 33/54 (61.1%) high-grade LDAVFs (Cognard IIb–V, *p* = 0.776).

A significant association was found between the endovascular technique and angiographic outcome according to Fisher’s exact test (*p* = 0.001). In detail, TV coil embolization more frequently yielded a complete occlusion of LDAVF (*n* = 11/14, 78.6%) when compared to TA liquid embolization (13/49, 26.5%).

### Radiation Dose and Fluoroscopy Time

**Fig. 2:**
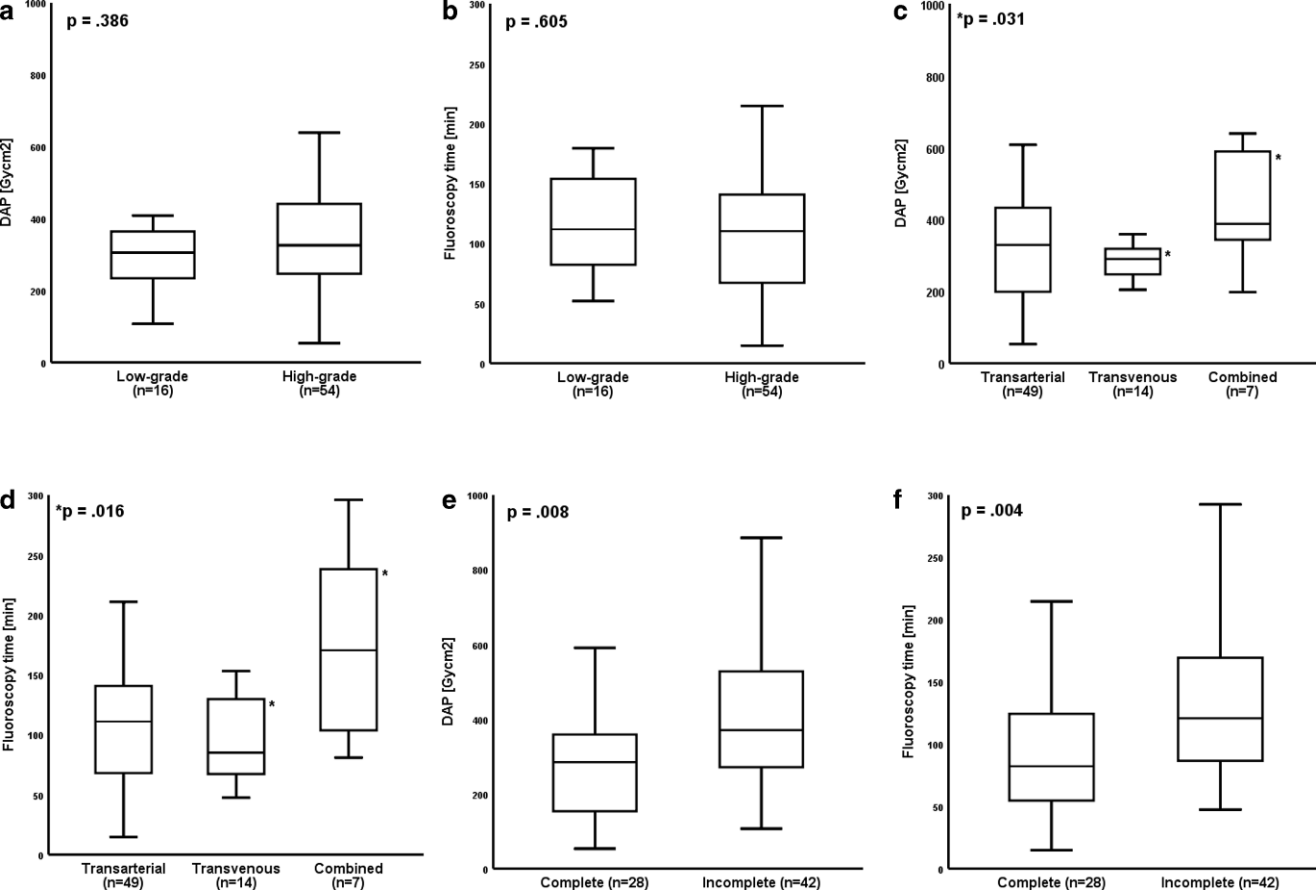
DAP and fluoroscopy time with respect to the Cognard classification (low-grade = I + IIa, high-grade = IIb–V), different endovascular techniques and angiographic outcome. Values shown as median (25%; 75% percentile). *DAP* dose area product

**Fig. 3:**
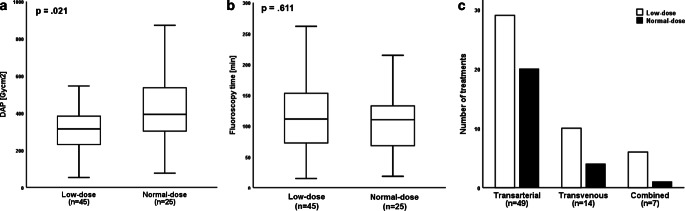
Impact of the DSA protocol on DAP and fluoroscopy time, and association with the endovascular technique. **a** LD protocol yielded a significantly lower DAP when compared to a ND protocol (**a**; *p* = 0.021), while the median fluoroscopy time was statistically equal between groups (**b**; *p* = 0.611). The interventional neuroradiologists preferentially decided for the LD protocol independent of the endovascular technique (**c**). *DAP* dose area product, *DSA* digital subtraction angiography, *LD* low-dose, *ND* normal-dose

Dosimetry results are presented in Table 2 and Figs. 2 and 3. With respect to the entire study population (*n* = 70), the median total DAP and fluoroscopy time were 325 Gy cm^2^ (245; 414 Gy cm^2^) and 110min (68; 142min), respectively. With respect to the annual trend of radiation dose within the study period, we observed a median total DAP of 491 Gy cm^2^ in 2014 (*n* = 9 patients), 260 Gy cm^2^ in 2015 (*n* = 9), 373 Gy cm^2^ in 2016 (*n* = 7), 287 Gy cm^2^ in 2017 (*n* = 19), 324 Gy cm^2^ in 2018 (*n* = 12), and 320 Gy cm^2^ in 2019 (*n* = 14).

**Table 2:** DAP and fluoroscopy time regarding the Cognard grade, endovascular technique, angiographic outcome, and DSA protocol in 70 minimally invasive treated patients with LDAVF

**Cognard grade**	**I** **(*n*** **=** **12)**	**IIa** **(*n*** **=** **4)**	**IIb** **(*n*** **=** **17)**	**III** **(*n*** **=** **10)**	**IV** **(*n*** **=** **26)**	**V** **(*n*** **=** **1)**
*DAP [Gy cm*^*2*^]	328 (216; 367)	290 (152; 806)	372 (282; 561)	184 (79; 283)	373 (283; 447)	101
*FL time [minutes]*	108 (71; 155)	112 (90; 128)	133 (77; 237)	78 (29; 125)	105 (67; 121)	54
**Cognard grade**	**Low-grade (I** **+** **IIa)**	**High-grade (IIb–V)**	**–**	***P*-value**		
*DAP [Gy cm*^*2*^]	305 (216; 367)	326 (245; 451)	**–**	*p* = 0.386		
*FL time [minutes]*	112 (82; 154)	110 (66; 141)	–	*p* = 0.605		
**Endovascular technique**	**TA liquid embolization** **(*n*** **=** **49)**	**TV coil embolization** **(*n*** **=** **14)**	**Combined TA/TV (*n*** **=** **7)**	***P*-value**		
*DAP [Gy cm*^*2*^]	329 (193; 434)	290 (246; 328)	388 (315; 639)	TA vs. TV: *p* = 0.261		
				TA vs. Combined: *p* = 0.177		
				**TV vs. Combined: *p*** **=** **0.031**		
*FL time [minutes]*	111 (66; 141)	85 (67; 130)	170 (96; 262)	TA vs. TV: *p* = 0.372		
				TA vs. Combined: *p* = 0.078		
				**TV vs. Combined: *p*** **=** **0.016**		
**Angiographic outcome**	**Complete occlusion (*n*** **=** **28)**	**Incomplete occlusion** **(*n*** **=** **42)**	**–**	***P*-value**		
*DAP [Gy cm*^*2*^]	285 (148; 359)	371 (271; 530)	**–**	***p*** **=** **0.008**		
*FL time [minutes]*	82 (54; 127)	121 (85; 170)	**–**	***p*** **=** **0.004**		
**DSA protocol**	**LD** **(*n*** **=** **45)**	**ND** **(*n*** **=** **25)**	**–**	***P*-value**		
*DAP [Gy cm*^*2*^]	315 (214; 386)	393 (287; 542)	**–**	***p*** **=** **0.021**		
*FL time [minutes]*	111 (70; 154)	110 (64; 133)	**–**	*p* = 0.611		

DAP and fluoroscopy time regarding the Cognard grade, endovascular technique, angiographic outcome, and DSA protocol in 70 minimally invasive treated patients with LDAVF. DAP and FL time values are provided as median (25%; 75% percentile). Pair-wise comparison of DAP and FL time with Bonferroni correction was performed using the Mann Whitney *U*-test. Significant values in bold

*DAP* dose area product, *DSA* digital subtraction angiography, *FL* fluoroscopy, *LD* low dose, *LDAVF* lateral dural arteriovenous fistula, *ND* normal dose, *TA* transarterial, *TV* transvenous

The highest median dosimetry values were calculated for LADVF Cognard grade III (DAP 184 Gy cm^2^; fluoroscopy time 133 min). When comparing low-grade (I + IIa) with high-grade LDAVF (IIb–V), median dosimetry values were not significantly different (Fig. 2a: DAP, *p* = 0.386; Fig. 2b: fluoroscopy time, *p* = 0.605).

With respect to the endovascular technique, the lowest dosimetry values were observed for TV coil embolization. In detail, both median total DAP and fluoroscopy time were significantly lower when compared to a combined TA/TV technique (Fig. 2c: DAP 290 versus 388 Gy cm^2^, *p* = 0.031; Fig. 2d: fluoroscopy time 85 versus 170 min, *p* = 0.016). Regarding TA liquid embolization (*n* = 49), we found a significant positive correlation between the number of treated arterial feeders and both DAP (r_s_ = 0.367; *p* = 0.010) and fluoroscopy time (rs = 0.295; *p* = 0.040). In patients treated by TV coil embolization (*n* = 14), no significant association was found between number of coils and the dosimetry parameters (DAP: r = 0.463, *p* = 0.096; fluoroscopy time: r = −0.362, *p* = 0.203).

Considering the angiographic outcome, median total DAP and fluoroscopy time was 285 Gy cm^2^ and 82 min for patients with complete, and 371 Gy cm^2^ and 121 min for patients with incomplete occlusion of LDAVF (Fig. 2e, *p* = 0.008 and Fig. 2f, *p* = 0.004, respectively).

Regarding the DSA acquisition type, a LD and ND protocol was applied in 45/70 (64.3%) and 25/70 (35.7%) patients, respectively, yielding a significantly lower median total DAP in the former group (Fig. 3a; 315 versus 393 Gy cm^2^, *p* = 0.021). In detail, median values of DAP_DSA_ and DAP_fluoroscopy_ were 230 and 85 Gy cm^2^ for the LD group as well as 325 and 68 Gy cm^2^ for the ND group. Median FL time was statistically equal between groups (Fig. 3b; *p* = 0.611). Independent of the endovascular technique, the treating physicians preferentially decided for the LD protocol (Fig. 3c; TA: *n* = 29/49, 59.2%; TV: 10/14, 71.4%; combined: 6/7, 85.7%).

The number of DSA acquisitions was not significantly different between groups (Cognard grade, endovascular technique, angiographic outcome, DSA protocol: *p*  0.05, each).

## Discussion

This retrospective single-center study provides detailed dosimetry data for the endovascular treatment of intracranial LDAVF in 70 patients between 2014 and 2019. In particular, we believe that the strict selection criteria (e.g., exclusion of other intracranial fistulae, such as carotid-cavernous fistula [17] or anterior cranial fossa DAVF [18]) as well as the dedicated consideration of both the Cognard grade and endovascular technique (TA liquid embolization with EVOH and/or TV coil embolization) is unique and may therefore be valuable in order to introduce novel DRLs in the field of interventional neuroradiology considering the paramount impact of the Euratom Basic Safety Standards directive [19].

For establishment of DRLs, which are defined as the 3rd quartile of the distribution of the DRL quantity, the International Commission on Radiological Protection (ICRP) 135 publication requires utilization of several dosimetry parameters such as DAP and fluoroscopy time [20]. As a consequence, the 3rd quartile of DAP, which represents a subsidiary dimension for X‑ray energy delivered to the patient [21], is commonly reported in neurointerventional studies [7–15]. In the present study, the median DAP and fluoroscopy time of the whole study population (*n* = 70) was 325 Gy cm^2^ and 110 min with a 3rd quartile value of 414 Gy cm^2^ and 142 min, respectively. Regarding the literature, to date merely few neurointerventional studies reported dosimetry values for intracranial DAVF treatment [13–16]. The median DAP in these studies ranged between 150 and 360 Gy cm^2^; in addition, the 3rd quartile either clearly exceeded our measured values (e.g., Kien et al. 726 Gy cm^2^) [14] or was not reported at all [16]. Moreover, the aforementioned studies neither yielded information concerning the dedicated type of intracranial DAVF nor the applied endovascular approach.

In this study, when subdividing the study cohort into low-grade and high-grade LDAVFs, both median DAP and fluoroscopy time were statistically equal between groups. These results suggest that dosimetry in endovascular LDAVF treatment may be independent of the Cognard grade. Instead, according to our data the endovascular technique significantly influences the radiation dose. In detail, we calculated significantly lower median values for LDAVF patients treated by TV coil embolization when compared to a combined TA/TV treatment (DAP 290 versus 388 Gy cm^2^; fluoroscopy time 85 versus 170 min). Also, the median values were lower in the TV coiling group when compared to TA liquid embolization (DAP 329 Gy cm^2^, fluoroscopy time 111 min), even though these differences did not reach statistical significance. Moreover, TV coil embolization commonly yielded a complete LDAVF occlusion, which in turn resulted in significantly lower median values when compared to patients in whom the fistula was not completely occluded (DAP 285 versus 371 Gy cm^2^; fluoroscopy time 82 versus 121 min).

In general, a comparably lower radiation dose in TV coil embolization is reasonable when envisioning the procedural workflow of endovascular LDAVF treatment. In Cognard grades I–IIb fistulae, TV access to the transverse and/or sigmoid sinus with subsequent therapeutic coil occlusion of the fistula-harboring sinus segment is commonly technically less sophisticated and succeeds more quickly when compared to the superselective catheterization of one or more arterial fistula feeders and time-consuming fluoroscopically-guided EVOH injection, except in the case of a highly stenotic and/or compartmentalized sinus [22, 23]. The significantly positive correlation between the number of arterial feeders treated by liquid embolization and both DAP and fluoroscopy time measured in this study underlines this statement. In contrast, the number of transvenously applied coils had no significant effects on radiation doses.

However, due to the nature of LDAVF the choice of the respective endovascular treatment is dependent on several morphologic and hemodynamic factors, such as amount and superselective accessibility of arterial feeders, the Cognard grade (sinus involvement only in grades I–IIb), patency of the respective sinus segment, and kind of venous filling (e.g., antegrade or retrograde flow within the vein of Labbé) [22]. For example, TV coil occlusion of a still patent fistula-harboring sinus segment alone is frequently not possible, since the subsequent hemodynamic alterations may yield hazardous venous congestion possibly resulting in intracranial hemorrhage. As a consequence, LDAVFs are commonly primarily treated via TA liquid embolization whenever technically feasible [2, 22–26]. Available embolic agents include particles, coils, ethanol, N‑butyl cyanoacrylate glue, and EVOH [22]. In this study, exclusively EVOH (Onyx® or Squid®) was used for TA LDAVF embolization, as previously published EVOH data suggested higher cure rates when compared to alternative embolic agents [22, 24, 26]. With respect to the angiographic outcome, LDAVFs embolized by TA access were downgraded in the majority of cases in comparison to a definitive occlusion in most patients undergoing TV coil embolization, which was clearly in the range of previously reported data [2, 27, 28].

Regarding radiation dose optimization in the field of interventional neuroradiology, several techniques have been proposed in recent years [7, 8, 12, 16, 29] in order to reduce the potential risk of deterministic radiation effects particularly when considering complex and time-consuming interventions and/or the necessity of multiple sessions [25, 26]. In this study, a LD DSA protocol, which was predetermined by the manufacturer, yielded a 20% reduction of the median total DAP when compared to a ND protocol (315 versus 393 Gy cm^2^). Disregarding fluoroscopy, because it was equal in both the LD and ND group, LD DSA mode allowed a dose reduction of approximately 30% (median 230 versus 325 Gy cm^2^). The positive impact of this dedicated DSA protocol on radiation dose was previously also demonstrated by Forbrig et al. who reported a 43% reduction of DAP in endovascularly treated patients with unruptured intracranial aneurysms [7]. Furthermore, van der Marel et al. reported a 37.6% reduction of DAP in endovascular treatment of intracranial DAVF when utilizing another angiography system with a dedicated dose reduction platform, which was not yet installed at our institution within the study period [16]; however, their sample size was low (normal dose *n* = 7; low dose *n* = 3), thus generalizability remains limited as intracranial DAVFs represent a heterogeneous spectrum of cerebrovascular diseases. In this context it is worth mentioning that according to the as low as reasonably achievable (ALARA) principle the maximum achievable dose reduction in DAVF treatment is limited as a reduced tube output may in turn substantially increase image noise, which can be hazardous particularly during TA injection of liquid embolic agents.

The results of the present study have to be evaluated in light of several limitations, as data were collected retrospectively from one neurovascular center. First, only one angiographic system from a single manufacturer (Axiom Artis, Siemens Healthineers) was used for neuroradiological interventions and data were only collected for the common subgroup of intracranial lateral DAVF. Hence, our dosimetry measurements can neither be generalized for other angiography suites, particularly those of the newest generation which contain additional techniques, probably enabling further reduction of radiation dose, nor the entire spectrum of intracranial DAVFs. Second, angiographic protocol settings were slightly variable (e.g., frame rate of DSA) depending on the respective physicians’ preference. Third, the following parameters were not collected: air kerma product and peak skin dose measured by thermoluminescent dosimeters. Instead, we decided to analyze the DAP because it is part of the automatically generated dose report. Therefore, it is easily accessible for every operator and can be used as a valid estimate for the applied radiation dose for each individual patient. We are aware that the DAP is only a surrogate parameter for the tube output and not equal to the exact effective patient dose; however, in analogy to the dose length product in computed tomography, the DAP in angiography is a clinically established and well accepted dose indicator. Finally, data of potentially conducted repeated/multiple sessions at other neurovascular sites (in patients in whom the LDAVF was only downgraded but not completely occluded) are missing, disabling report of cumulative doses which might be associated with the respective Cognard grade; however, we believe that our results may serve as a dosimetry baseline dataset in the field of neurointerventional LDAVF treatment, since comparable studies have not yet been published.

## Conclusion

This study provides detailed dosimetry data for the endovascular treatment of intracranial LDAVF, which may be substantial for definition of novel DRLs. The DAP and fluoroscopy time were dependent on the endovascular technique, with the lowest values for TV coil embolization. A higher number of arterial feeders treated by liquid embolization (EVOH: Onyx®, Squid®) was associated with an increased DAP and fluoroscopy time, whereas the number of transvenously applied coils did not substantially alter radiation dose. Complete LDAVF occlusion was associated with TV coil embolization, thus yielding a lower DAP and fluoroscopy time when compared to LDAVF downgrading. A low-dose DSA protocol yielded a 20% reduction of radiation dose. Apart from various subjective parameters (e.g., institutional preferences and preconditions), this technical approach enables a significant dose reduction in interventional neuroradiology without compromising the capability of individual patient treatment. Prospective multicenter studies are warranted, including evaluation of modern angiography suites with novel dose reduction techniques, alternative DSA and fluoroscopy settings (e.g., reduced frame rates), and collection of cumulative doses in patients undergoing multiple treatments.
